# Supplementary material for: Abdominal ultrasound in the diagnostic work-up of visceral leishmaniasis and for detection of complications of spleen aspiration
Source: PLoS Negl Trop Dis. 2021 Feb 16;15(2):e0009107. doi: 10.1371/journal.pntd.0009107 (PMC7935305; doi:10.1371/journal.pntd.0009107)
Supplement: S2 Table — (DOCX) [file pntd.0009107.s002.docx]

**Supplementary table 2: Abdominal ultrasound finding in VL suspects at the Leishmaniasis Research and Treatment center, Gondar, Ethiopia (Oct 2017 – Dec 2018; N=242)**

| Ultrasound finding | HIV patient (N/%) | Non HIV patient |
| --- | --- | --- |
| Ascites | | |
| Yes | 2 (4.0) | 26 (14.0) |
| No | 46 (96) | 164 (86) |
| Plural effusion | | |
| Yes | 1 (2.0) | 5 (3) |
| No | 48 (98.0) | 184(97.0) |
| Splenic echo texture | | |
| Normal | 47 (96.0) | 184(97.0) |
| Abnormal | 2 (4.0) | 6 (3.0) |
| Splenomegaly ^a^ | | |
| Yes | 48 (100.0) | 188 (99.0) |
| No | 0 (0.0) | 2 (1.0) |
| Splenic Nodule | | |
| Yes | 3 (6.0) | 5 (3.0) |
| No | 46 (94.0) | 184 (97.0) |
| Lymphadenopathy | | |
| Yes | 0 (0.0) | 2(1) |
| No | 49 (100.0) | 188 (99.0) |
| Hepatomegaly ^b^ | | |
| Yes | 36 (73.0) | 139 (75.0) |
| No | 13 (27.0) | 47 (25.0) |
| Liver echotexture | | |
| Normal | 46 (94.0) | 180 (95.0) |
| Abnormal | 3 (6.0) | 10 (5.0) |
| Liver focal lesion |  |  |
| Yes | 0 (0.0) | 4 (2.0) |
| No | 49 (100.0) | 186 (98.0) |
| Renal echotexture |  |  |
| Normal | 48 (100.0) | 183 (97.0) |
| Abnormal | 0 (0.0) | 6 (3.0) |
| Renal focal lesion |  |  |
| Yes | 0 (0.0) | 2 (1.0) |
| No | 49 (100.0) | 188 (99.0) |

^a^ Splenomegaly, spleen size measured from pole to pole >12 cm

^b^ Hepatomegaly, liver measured longitudinally right lobe diameter size > 15cm
