# Supplementary material for: Abdominal ultrasound in the diagnostic work-up of visceral leishmaniasis and for detection of complications of spleen aspiration
Source: PLoS Negl Trop Dis. 2021 Feb 16;15(2):e0009107. doi: 10.1371/journal.pntd.0009107 (PMC7935305; doi:10.1371/journal.pntd.0009107)
Supplement: Attachment — Submitted filename: Cover letter response with point by point reponse.docx [file pntd.0009107.s004.docx]

Date: 20 October 2020

To Editor-in-Chief, PLOS NTD

**RE: Abdominal ultrasound in the diagnostic work-up of visceral leishmaniasis and for detection of complications of spleen aspiration**

Dear Sir/Madam,

We thank you very much for considering our paper and for the reviewers’ comments. We have attempted to address all these comments and have revised our paper accordingly. In addition, we have provided a point-by-point response below.

While hoping that these changes would meet with your favourable consideration, we hold ourselves at your entire disposition for any further information or other changes you might require.

Yours Sincerely,

On behalf of all authors

Rezika Mohammed, MD

Department of internal medicine,

University of Gondar, Gondar Ethiopia

Reviewer's Responses to Questions

Reviewer #1: I think this is a prospective study, which was very well conceived, executed and described. I would recommend accepting it with minimal revisions.

**Reply: thank you for your encouraging words.**

Reviewer #3: - I suggest to assess the differences in proportion of the variables in the VL and non-VL patients with statistical tests. The authors should report the p-values in table 1, table 2 and table 4

**Reply: this has been added now. While conducting these tests, we observed one patient was misclassified. We have corrected that now.**

Reviewer #1: Comments:

What was not clear to me is on which criteria the authors decided whether to do a SA or a bone marrow aspirate. Maybe this could be better clarified in the methods section.

**Reply: this was been added on page 6 # line #128-130**

Minor comments:

L34: remove bracket:

**Reply: Modified as suggested**

L120 convenience sampling method?

**Reply: All eligible patients were consecutively enrolled. This has now been clarified.**

L130 Patients who could

**Reply: the statement is modified now**

L123-136 should be written in past tense as the rest of the section:

**Reply: Modified as suggested**

L126 what was the descision criteria to do BMA vs SA?

**Reply: This was been added on page # 6line #128-130**

L186 was more common –

**Reply: Modified as suggested**

L 323 I would not say US is highly subjective, I think that is an anachronism and true for almost every medical examination – from listening to the sound of a heart to looking at a CXR. I would suggest “examiner- and training-dependent”.

**Reply: Modified as suggested**

L334 The last recommendation to preform post intervention US sounds strange in light of the fact that injuries were rare and none of the detected injuries led to consequences. I still understand (and agree) that it should be done- but maybe the statement could be tuned down.

**Reply: We have tried to phrase this more carefully. Line #379-382**

**“The occurrence of splenic injury after SA emphasizes for close observation after the procedure and a careful risk-benefit assessment. If available, US post-aspiration could further add to the safety of the procedure for patients undergoing SA for detection of spleen injuries.”**

Table 1 spleen size median (IQR) vs Yes (cm below costal margin)

**Reply: We have clarified this now.**

Table 2 Anemia – is this in % or absolutes?

**Reply: It is g/dL (please see the footnote)**

rK39 data is confusing – what are absolutes what are percent and what are the percentages on: I would suggest to use the percentage of those tested instead of percentage of all patients. I think table 2 needs revision regarding the units, %, IQR…

**Reply: we agree this table was not entirely clear and have modified.**

Reviewer #3: I suggest this article could be improved by some revisions:

There are some typing errors (capital letters, punctuation…);

**Reply: we have carefully revised the manuscript and have done some editorial changes**.

- In the “Study procedures” section I suggest to indicate which kind of needle has been used for the spleen aspiration (in particular the gauge of the needle). I suggest to specify if US-guided spleen aspiration has been performed;

**Reply: US was done blindly (not US guided) using a 21 gauge needle. We have added this information now (line # 130 & 131).**

- In the “data collection and analysis” section the authors mentioned that “radiologists” performed all the US. How many radiologists? How much experience have they in abdominal US? Interpretation and variability of the results could be influenced by these specifications;

**Reply: Four radiologists were involved in the study. Three of them were at the end of their second year radiology residency course experienced with doing more than 500 abdominal US. One senior radiologist was regularly doing quality checks of the US done. Added in line#172-175**

- I have noticed that in this population there was a high proportion of HIV-positive patients. The most part of the US abnormalities have been evaluated (particularly splenic nodules and abdominal lymphadenopathies) are frequently detected also in HIV-patients with abdominal TB. In such a setting with a high prevalence of HIV/TB co-infection as Ethiopia, it would be possible to deal with abdominal TB very often. How did the authors definitively exclude abdominal TB in patients with these US abnormalities? There was any VL-patient with splenic nodules or abdominal lymphadenopathies who had a TB co-infection? I suggest to comment these aspects in the discussion;

**Reply: We agree this merits some more elaboration. While it was already somehow addressed in the discussion, we have elaborated more on this now in the result section (line # 236 241)**

- I suggest to describe US features of splenic nodules and hepatic lesions (micro-/macro-nodules, hypo/hyper-echoic, single/multiple/miliary…);

**Reply: We agree such information might be useful. Splenic nodules were hyperechoic, small, multiple and non caseating. No specific US features/ description seen for VL suspects in this study. . However, description fo the hepatic nodule is unfortunately not available.**

- I suggest to assess the differences in proportion of the variables in the VL and non-VL patients with statistical tests. The authors should report the p-values in table 1, table 2 and table 4;

**Reply: added as suggested. While conducting these tests, we observed one patient was misclassified. We have corrected that now.**

- In table 5, point 3: I suppose the authors would mean “hypo-echoic lesion”;

**Reply: thank you, indeed. Modified as suggested.**

- Line 219: I suppose the authors would refer to longitudinal hepatic right lobe diameter ≥ 15 cm.

**Reply: thank you. Modified in study procedure section of the manuscript and on table 3 and 4.**

Reviewer #1: This is a first systematic study on the use of ultrasound in VL in an endemic setting. I is therefore novel and scientifically significant.

**Reply: Thank you for the encouraging words**

Reviewer #2: The authors present ultrasound and clinical data from a prospective study in patients with suspected visceral leishmaniasis (VL) in Ethiopia. The study cohort comprises around 400 patients with half of them having confirmed VL. Abdominal ultrasound, performed by the radiology department, was evaluated as diagnostic and follow-up test for VL and as a tool to detect splenic injury after diagnostic spleen aspiration. Additionally, the study evaluated severity of pain associated with spleen aspiration. The study addresses an important topic with clinical relevance and is generally well presented. However, there is considerable potential for improvement of the manuscript by elaborating more on the case definitions and co-infections (see comments below).

My main concerns with the data are the case definitions:

First, in non VL patients RDT was positive in 60% and diagnoses other than VL are reported for 23% only. How sure are you having excluded VL?

**Reply: Thank you for this important point. We agree that there could be missed VL cases in the non-VL group (tissue aspirate negative), but we think this to be very rare in our setting for the following reasons**.

**The experience with the rK39 RDT in Ethiopia is not very good, even more so in HIV patients, with suboptimal sensitivity and specificity. In our setting, at least at in the Leishmaniasis Research and Treatment setting, VL diagnosis is essentially based on tissue aspiration.**

**For tissue aspirate negative patients with no alternative diagnosis and still a high clinical suspicion of VL, in some instances, clinicians might still decide to provide VL treatment in tissue-aspirate negative patients if clinical suspicion is very high. While we did not collect information on this, based on our experience, this is very exceptional in our setting.**

**We nevertheless agree that some misclassification can not be ruled out and have acknowledged that as an important study limitation.**

Second, 18% of VL patients had concomitant infections of which some are known to present with similar sonographic features as VL. As detailed data for these concomitantly infected patients are not presented separately the reader does not know if e.g. splenic nodules occurred in VL/TB co-infected patients and hence etiology of splenic nodules (TB or VL) remains unclear. Presenting/describing sonographic data for co-infected patients (or exclusion of co-infected patients) appears very important not only for a more accurate calculation of the diagnostic value but also for interpretation of follow-up data. Follow-up US after VL treatment did not lead to a resolution of all findings, there was even occurrence of some new findings.

**Reply: We agree such information might be useful to add. We have now provided US data for HIV pos and HIV neg patients separately in the supplementary information**. **Findings are generally very similar.**

Did co-infected patients receive treatment for their co-infections in parallel?

**Reply: indeed, identified co-infections would be treated once diagnosed.**

If I understand correctly, patients were not systematically investigated for co-infections like TB (especially as 18% were HIV-infected and  VL has been discussed as additional risk factor for TB). Therefore, even if you exclude co-infected patients from your cohort there remains the limitation of possible undetected co-infection. This should be highlighted in the limitations/discussion.

**Reply: We agree TB diagnosis in the setting of VL remains challenging and there could indeed be some cases of TB missed in this population. This is included as one of the study limitations. We have now also provided US data for HIV pos and HIV neg patients separately in the supplementary information. Findings are generally very similar.**

Given the considerable ratio of HIV infected patients in the VL (and non-VL group) I would be interested if you see any difference in US findings in HIV-infected versus non-infected (in TB HIV-co-infection has a significant impact on US findings).

**Reply: thank you, valid point. We have now provided US data for HIV pos and HIV neg patients separately in the supplementary information.**

Please find further comments below:

Abstract:

1. The abstract should include the study site and study period.

**Reply: Modified as suggested.**

Introduction:

2.    As US was also evaluated for follow-up, consider adding a short mention of the need/value for a follow-up test in VL.

**Reply: mentioned on line 98.**

Methods:

3.    As the paper is mainly about ultrasound, ultrasound methodology should be described in more detail in the methods section. Reference 20 is a Book review of the original text with the same title; an original reference should be used (if needed). As splenomegaly and hepatomegaly are major findings, sonographic definitions of splenomegaly and hepatomegaly should be provided in the text (how were measurements performed, what were the cut-offs for definitions; comparable to the footnote to Table 3 with addition on how the liver was measured). Same for lymphadenopathy, how was abdominal lymphadenopathy defined (size of lymph nodes in what axis). Was there a definition of abnormal echotexture of organs?

**Reply: Definitions are added in line # 159- 164. Reference modified.**

Results:

4.    192 VL suspects were parasitologically confirmed; the remainder (201) were classified non-VL cases. As in the methods you mention “If no other diagnosis can be identified treatment for VL might be initiated based on RDT results” – and in Table 1 only 22.5% of non-VL patients had a concomitant infection and 58% had a positive RDT, please clarify on the final diagnoses of the 77.5% patients without VL and without other infections. Did any of these patients receive VL treatment as outlined in the methods? If so, case definitions and groups should be reconsidered. If not, please make this clear in the manuscript and comment on how confident you are that VL was excluded in these patients.

**Reply: We acknowledge the issue of misclassification of cases and non cases and we clarify this in the limitation section. Please see our reply above to this point.**

5.    In Figure 1 adding the 174 spleen aspirate positive patients to the 23 bone marrow aspirate positive patients results in 197 VL positive patients; thanks for clarifying why this figure differs from the 192 confirmed patients in the text.

**Reply: thank you for pointing this out. We have corrected it.**

6.    It would be very valuable to add more sonographic information on the splenic nodules. What was their echogenicity, number, size etc. This is particularly interesting as splenic nodules do also occur in other infectious diseases (e.g. TB) and to date it is unknown if these look identical or if there may be distinctive features.

**Reply: Splenic nodules were hyperechoic, small, multiple and non caseating. No specific US features/ description seen for VL suspects in this study.**

7.    Similarly, more detailed information on sonographic lymphadenopathy would be valuable (e.g. location, size, number, echogenicity).

**Reply: On abdominal US lymphadenopathy were considered significant when the size of the LAP was > 1cm. However, a detailed description of the lymphadenopathies is not available.**

8.    Similarly, addition of more sonographic description of the abnormality of organ echotexture for spleen, liver, kidneys would be valuable.

**Reply: This is described on lines #162 & 164.**

9.    Similarly, some more information on the quantity of pleural effusion would be valuable.

**Reply: Thank you, valid point. This information was however not collected.**

10.    Was follow-up US only done in VL cases? If non-VL cases also received a follow-up scan it would be very interesting to see the evolution of findings in the absence of VL treatment.

**Reply: this was not done for non-VL cases; VL cases remain in the VL research and treatment center; non-VL cases are referred to other departments of the hospital.**

11.    To understand the sonographic follow-up data and related dynamics it is very important that you add data on the duration of treatment or time frame until follow-up US. Currently the reader cannot know if this is a few days, weeks or months.

**Reply: Most patients received SSG and paromomycin combination therapy. The median duration of treatment was 17 days (IQR, 17-28). We have added that in the text.**

12.    In non VL patients – how where findings of liver and spleen lesion interpreted? What was the (most likely) etiology? Consider describing separately diagnoses (and course) of patients with liver or spleen lesions to better understand their possible etiology (in VL and non-VL patients).

**Reply: Thank you for this valid point. Unfortunately, such information was not collected. We have added the lack of detailed information on the non-VL group as a study limitation**.

Discussion

13.    The discussion would benefit from a more critical discussion of the study’s methods and limitation (in line with the comments above). A further limitation that should be mentioned is that a linear probe was not used in all patients for assessment of the spleen; a higher detection rate of splenic nodules is achieved with linear probes and therefore in this cohort some may have had undetected nodules. The following reference shows the diagnostic difference between linear and convex probe for splenic nodules:

Bernabeu-Wittel, M., J. L. Villanueva, J. Pachon, A. Alarcon, L. F. Lopez-Cortes, P. Viciana, F. Cadaval and A. Talegon (1999). "Etiology, clinical features and outcome of splenic microabscesses in HIV-infected patients with prolonged fever." Eur J Clin Microbiol Infect Dis 18(5): 324-329.

**Reply: We have integrated several points raised by the reviewer in the discussion. We agree with the reviewer on the advantages of linear probes. Fortunately, we used a linear probe for splenic nodules, (please see line 154).**

14.    If indeed ultraound findings in VL are rare, the discussion could also include discussion of a potential value of TB ultrasound to detect concomitant TB in VL given the difficulty of confirming TB in resource-limited settings and in HIV-infected patients and particularly as concomitant TB is a risk factor for poor outcomes in VL/HIV co-infected patients.

**Reply: we fully agree with this point. We have expanded on this now in the discussion as an area for future research (line 333-337).**

Reviewer #3: I suggest this article is instructive and well written. I have appreciated the authors’ effort to improve US in the work-up of visceral leishmaniasis. I believe that the large sample size and the presence of a control group are the strengths of this article.

**Reply: thank you for your kind words.**
