# Supplementary material for: Abdominal ultrasound in the diagnostic work-up of visceral leishmaniasis and for detection of complications of spleen aspiration
Source: PLoS Negl Trop Dis. 2021 Feb 16;15(2):e0009107. doi: 10.1371/journal.pntd.0009107 (PMC7935305; doi:10.1371/journal.pntd.0009107)
Supplement: Attachment — Submitted filename: Response to reviewer_US.docx [file pntd.0009107.s005.docx]

Date: 26 December 2020

To Editor-in-Chief, PLOS NTD

**RE: Abdominal ultrasound in the diagnostic work-up of visceral leishmaniasis and for detection of complications of spleen aspiration**

Dear Sir/Madam,

We thank you very much for considering our paper and for the reviewers’ comments. We have attempted to address all comments and have revised our paper accordingly. In addition, we have provided a point-by-point response below.

While hoping that these changes would meet with your favourable consideration, we hold ourselves at your entire disposition for any further information or other changes you might require.

Yours Sincerely,

On behalf of all authors

Rezika Mohammed, MD

Department of internal medicine,

University of Gondar, Gondar Ethiopia

Reviewer's Responses to Questions

Reviewer #3: Table 2, row "Positive rK 39 RDT", column "non-VL": correct (67.7) not (677)

**Reply: Comment accepted and corrected**

Reviewer #2: The authors have very much improved their manuscript by the revisions suggested by the reviewers and I believe this is an important work for the concerned readers.

I have two remaining points, both referring to the splenic nodules seen by ultrasound - which constitute the most interesting sonographic findings in my view.

I had pointed to the importance that linear probes are used to detect splenic nodules (at least this is the case for splenic nodules due to TB). The authors replied that fortunately a linear probe was used, but in the methods it reads that a linear probe was only used to confirm e.g. splenic nodules. I therefore assume a linear probe was only used if splenic nodules were seen by convex probes. Therefore my first point remains true - if you don't use a linear probe for screening you will likely miss nodules in the spleen. It think this should be discussed (at least in the limitation sections) because reported prevalence of splenic lesions might have been an underestimate of the true prevalence.

I am surprised to read that all splenic nodules were hyperechoic because the literature mostly reports hypoechoic splenic lesions in VL. It would be very interesting if you could provide a characteristic ultrasound image of splenic nodules in a VL patient to accompany your article

**Reply: Thank you, we agree that we did not use the linear probe for screening of nodules. We modified the manuscript and add it as a limitation. Line # 371 and 372.**

**We agree that most infectious like VL related splenic nodules are mostly hypoechoic but in our case we found hyperechoic splenic nodules on US on the all our subjects ( three in VL and seven in Non- VL cases). Unfortunately we could not attach the ultrasound picture of these patients.**

**General editorial comment**

Your response to reviewers is duplicated. To avoid confusion, please remove your 'response to reviewers' from your cover letter.

**Reply: File renamed to avoid confusion**

Please provide an Author Summary. This should appear in your manuscript between the Abstract (if applicable) and the Introduction, and should be 150–200 words long. The aim should be to make your findings accessible to a wide audience that includes both scientists and non-scientists. Sample summaries can be found on our website under Submission Guidelines.

**Reply: This is well noted and was added between abstract and introduction. Line number #44**

We are unable to open your [Fig 1 US IN VL.tif] file. Please kindly revise as necessary and re-upload.

**Reply: The figure is uploaded again**

In the online submission form, you indicated that you need assistance with your Data Availability Statement. Please reply to us via return email so we can best address your concerns and remedy your Data Availability Statement. If the box for this was ticked in error, please update your submission form answer, and ensure your Data Availability Statement is compliant. All PLOS journals require data underlying the findings described in the manuscript to be freely available to other researchers, either in a public repository, or in the manuscript itself. This policy applies to all data except where public deposition would breach compliance with the protocol approved by your research ethics board. If your data cannot be made publicly available for ethical or legal reasons (e.g., public availability would compromise patient privacy), please explain your reasons by return email and your exemption request will be escalated to the editor for approval. Your exemption request will

be handled independently and will not hold up the peer review process, but will need to be resolved should your manuscript be accepted for publication. One of the Editorial team will be in touch if they require more information.

**Reply: the box was ticked by error, now submission is updated**
